# Landscape Elements and Hantaan Virus–related Hemorrhagic Fever with Renal Syndrome, People’s Republic of China

**DOI:** 10.3201/eid1309.061481

**Published:** 2007-09

**Authors:** Lei Yan, Li-Qun Fang, Hua-Guo Huang, Long-Qi Zhang, Dan Feng, Wen-Juan Zhao, Wen-Yi Zhang, Xiao-Wen Li, Wu-Chun Cao

**Affiliations:** * State Key Laboratory of Remote Sensing Science, Beijing, People’s Republic of China; † State Key Laboratory of Pathogen and Biosecurity, Beijing, People’s Republic of China; 1 These authors contributed equally to this article.

**Keywords:** Landscape elements, hemorrhagic fever with renal syndrome (Apodemus type), remote sensing, GIS, research

## Abstract

Hemorrhagic fever with renal syndrome (HFRS) is an important public health problem in the People’s Republic of China, accounting for 90% of human cases reported globally. In this study, a landscape epidemiologic approach, combined with geographic information system and remote sensing techniques, was applied to increase our understanding of HFRS due to Hantaan virus and its relationship with landscape elements in China. The landscape elements considered were elevation, normalized difference vegetation index (NDVI), precipitation, annual cumulative air temperature, land surface temperature, soil type, and land use. Multivariate logistic regression analysis showed that HFRS incidence was remarkably associated with elevation, NDVI, precipitation, annual cumulative air temperature, semihydromorphic soils, timber forests, and orchards. These findings have important applications for targeting HFRS interventions in mainland China.

Hemorrhagic fever with renal syndrome (HFRS) is a zoonosis caused by different species of hantavirus (HV). It was first recognized in northeastern China in 1931 and has been prevalent in many other parts of China since 1955. At present, HFRS is endemic in 28 of 31 provinces of the People’s Republic of China, autonomous regions, and metropolitan areas and accounts for 90% of the HFRS cases reported globally (*1*). The disease has taken a heavy toll on the health of the Chinese people, having been responsible for 1.2 million symptomatic infections and 44,300 deaths from 1950 to 1997.

In China, HFRS is mainly caused by 2 HVs, i.e., Hantaan virus (HTNV) and Seoul virus (SEOV), each with a distinct rodent host. HTNV, which causes more severe disease, is carried by *Apodemus agrarius*. SEOV, which causes a less severe form of HFRS, is carried by *Rattus norvegicus*. A novel HV named Amur virus (AMRV) was identified recently in *A. peninsulae* from far eastern Russia and subsequently identified in a few patients from China (*2*,*3*). Another HV, designated as Soochong virus, was recently isolated from *A. peninsulae* in Korea and was described as an antigenically and genetically distinct HV species, which was monophyletic with AMRV but not with *A. agrarius*–associated HTNV (*4*). HVs are primarily transmitted from rodent host to human by aerosols generated by contaminated urine and feces and possibly by contaminated food or rodent bites (*5*,*6*).

Previous studies indicated that HFRS incidence seemed to be associated with environmental factors, including topography, hydrologic features, and rainfall. HFRS cases were mainly reported from areas <500 m above sea level and in the regions with very moist soil. HFRS cases were rarely reported in areas that were very dry or very wet (*7*–*10*).

Recently, we analyzed the distribution of HFRS cases in China based on geographic information system (GIS) spatial analysis (*11*) and found areas where the population had a high risk of acquiring the disease. That study demonstrated a new approach to integrating such tools into the epidemiologic study and risk assessment of HFRS.

Our objective for the current study was to identify the relationship between the incidence of HFRS due to HTNV and landscape elements by using the concepts of landscape epidemiology as well as GIS and remote sensing techniques. The major landscape elements considered in this study were elevation, normalized difference vegetation index (NDVI), precipitation, annual cumulative air temperature, land surface temperature (LST), soil type, and land use. The study focused on HFRS cases caused by HTNV only and restricted study sites to rural areas of the country and the areas with population density <1,000/km^2^.

## Methods

### Data Collection and Management

All the cases reported in mainland China from 1994 through 1998 were obtained from the National Notifiable Disease Surveillance System (NNDSS). NNDSS is supported by a special monitoring network and produces these data annually according to county, a political subdivision of a province, which usually contains several townships and has a population of ≈500,000 persons.

Because the number of cases was small and varied yearly in each county, we used the mean number of HFRS cases from each county from 1994 to 1998. All HFRS cases were coded according to geographic area (geo-coded) and matched to the corresponding polygon and its label point on a digital map of China by using the software ArcGIS 9.1 (ESRI Inc., Redlands, CA, USA). The NNDSS HFRS data do not differentiate HTNV from SEOV infections. The study was limited to the rural areas of the country and areas with population density <1,000/km^2^, to capture most, if not all, of the patients infected with HNTV.

Demographic data at the county level were obtained from the 1995 and 2000 censuses. To overcome difficulties due to changes in administrative boundaries, the vector map of the demographic data was converted to a raster map of the population with a 1-km grid (*12*). Based on the 1995 and 2000 maps for the population and the map of the administrative units, the average population of each county was calculated.

Remote sensing information was used to generate a digital elevation model (DEM) with a 1:100,000 scale. The elevation data obtained from DEM was transferred into a raster map with a 1-km grid (*12*). Based on DEM and the map of the administrative units, the average elevation of each county was calculated. Counties were then classified into 8 levels (meters above sea level): <100, 101–200, 201–500, 501–1,000, 1,001–1,500, 1,501–2,000, 2,001–3,000, and >3,000.

The NDVI was derived by the National Satellite Meteorological Center in China by using advanced, high-resolution radiometer (AVHRR) images. Monthly and annual NDVI in 1998 were calculated by using ERDAS Imagine 8.7 (Leica Geosystems Geospatial Imaging, LLC., Norcross, GA, USA) (*12*). Counties were classified according to 4 NDVI levels: <0.1, 0.101–0.2, 0.201–0.3, and >0.3.

The annual precipitation data were based on the average of the cumulative annual precipitation in China from 1994 to 1998, obtained from 700 weather stations (*12*). The inverse distance weighting (IDW) method was applied to interpolate and generate its raster map for annual precipitation with a 1-km grid (*13*,*14*). Annual precipitation values were divided into 4 levels: <400, 401–800, 801–1,200, and >1,200 mm. These levels corresponded to arid, semiarid, semihumid, and humid areas, respectively.

Air temperature data were obtained from 700 weather stations through the country from 1970 to 2001 (*12*). The IDW method was applied to interpolate and generate its raster map with a 1-km grid. The average daily temperature of each county was added to derive the annual cumulative air temperature, and it was divided into 5 temperature ranges: <1,600°C, 1,600°C–3,399°C, 3,400°C–4,499°C, 4,500°C–7,999°C, and >8,000°C. These levels represent frigid-temperate, mid-temperature zone, warm-temperate, semitropical, and tropical zones, respectively.

LST data at county level were also obtained from the monthly AVHRR 1998 data (*12*). LST values were divided into 5 levels: <28°C, 28°C–31°C, 32°C–34°C, 35°–37°C, and >37°C.

The soil types in the map were grouped into 12 categories, i.e., argosols, semiluvisols, caliche soils, arid soils, desert soils, skeletol primitive soils, semihydromorphic soils, hydromorphic soils, saline soils, anthrosols, alpine soils, and ferralisols. These categories are based on the Classification and Codes of Soil in China (*12*).

The types of land use in the map were categorized as paddy land, irrigated land/nonirrigated farmland, timber forest land, orchard land, sparse woods, bush, prairie and grassland, hilly/mountainous grassland, desert (desert, Gobi, cold desert), wetland, saline-alkali land, and bare land (*12*). The timber forest land is used to produce timber for building and furniture; orchard land produces fruits and raw materials for industry or for beverages and medicines, for example. The Gobi is a large desert region of southeast Mongolia and northern China, which consists mainly of series of shallow alkaline basins. Bush has been defined as land covered with dense vegetation or undergrowth.

### Data Analyses

To process the data for landscape elements at county level, we overlaid the map of administrative units on the raster map of each landscape element. The average elevation, NDVI, air temperature, LST, precipitation, area proportions with different type of soils, and land use were then calculated for each county by using ArcGIS 9. The average annual HFRS incidence of each county was calculated as well. Through the linkage of the 6-digit county geo-code, the incidence of HFRS at county level was displayed on the base map with administrative boundaries and then converted to a raster map, which was overlaid on the thematic maps of the landscape elements.

HFRS incidence was also calculated for each category of the landscape elements by overlaying maps of HFRS with the different thematic maps. For example, elevation was divided into 8 levels and then displayed on the map of elevation for the whole country. According to the area proportions of each level of elevation, the population and the number of HFRS cases at the county level were displayed as HFRS incidence data at each elevation level were then obtained.

Univariate analysis (χ^2^) was used to compare HFRS incidence across the different levels of each landscape element, including elevation, NDVI, precipitation, annual cumulative air temperature, and LSTl; odds ratios (ORs) were obtained by comparing the HFRS incidence of different categories of the landscape elements. To determine the associations between HFRS and soil type as well as land use, univariate logistic analysis was conducted, and ORs were computed by comparing counties where HFRS was found with non-HFRS–endemic counties. Through GIS, different thematic maps were also generated to facilitate graphic and spatial visualization of HFRS occurrence at the county level in China and geographic distribution of the different landscape elements (*15*).

Multivariate logistic regression analysis was then performed. The dependent variable was whether HFRS occurs; independent variables were landscape elements (elevation, NDVI, precipitation, annual cumulative temperature and LST, type of soil, and land use). Backward stepwise selection was performed with the criterion of p>0.05.The possible interaction between individual elements was considered.

Condition indexes and variance decomposition proportions were used to test colinearity among the independent variables and identify the sources of colinearity. When the condition index was >30, the independent variables had strong colinearity. If a large condition index is associated with variables that have variance decomposition proportions >0.5, these variables may be causing colinearity problems (*16*)*.*

## Results

**Figure 1:**
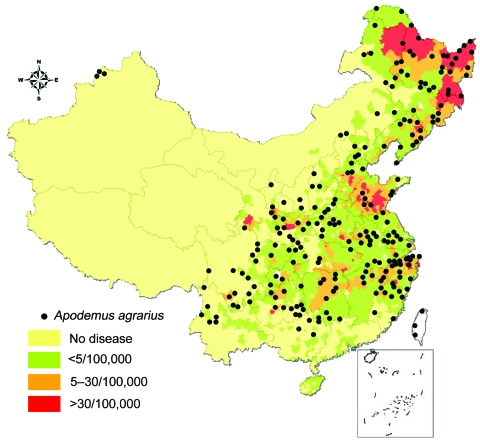
Geographic distribution of incidence of hemorrhagic fever with renal syndrome (HFRS) in the People’s Republic of China and relationship with capture points *of Apodemus agrarius.* Black dots show the capture points of *A. agrarius.* Internal borders indicate provinces.

**Figure 2:**
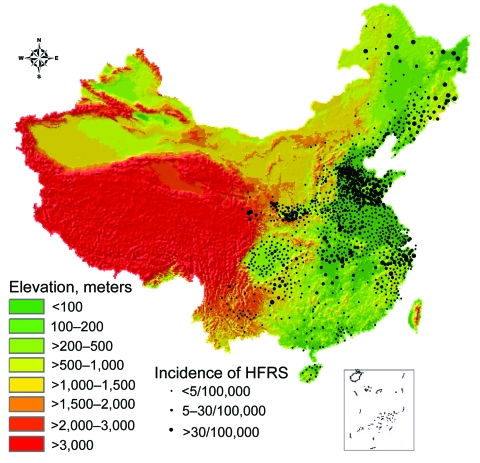
Topographic map of the People’s Republic of China, showing relationship between elevation and incidence of hemorrhagic fever with renal syndrome (HFRS).

The average HFRS incidence of each county in mainland China is displayed in Figure 2, with an overlaid map of *A. agarius* capture points (*17*). The top 6 incidence rates were 20.3, 18.9, 8.2, 7.7, 5.0, and 4.6/100,000 population in Heilongjiang, Shandong, Zhejiang, Hunan, Hebei, and Hubei Provinces, respectively. Approximately 70% of HFRS cases were reported from the above provinces. Only Xingjiang, Tibet autonomous regions, and Qinghai Province never reported any HFRS cases Figure 1.

HFRS incidence significantly declined as elevation increased (χ^2^ for trend test, p<0.001; Spearman correlation test r = –0.466, p<0.01). The highest incidence (7.3/100,000 population) was observed in areas with elevation of 100–200 m. No cases were reported in areas >3,000 m except in 3 counties of Gansu Province (XiaHe, Diebu, and Zhuoni). Approximately 86.4% HFRS cases occurred in areas with 0–500 m elevation in the eastern part of China and the Sichuan Basin (Figure 2).

HFRS incidence was 3–4× higher in areas with an NDVI 0.1–0.3 than in areas with NDVI <0.1 (Table 1). There were significant differences in HFRS incidence in regard to NDVI (df = 3, p<0.001). However, the peak incidence of 4.6/100,000 population was observed at an NDVI level of 0.2–0.3. These areas are mainly located in the eastern and middle part of China.

**Table 1:** HFRS incidence at different levels of landscape elements, China*

	Incidence (95% CI)†	p value‡	Odds ratio (95% CI)
Elevation, m			
<100§	3.48 (3.41–3.52)	–	1.00
100–200	7.29 (7.16–7.43)	<0.001	2.10(2.05–2.16)
201–500	5.17 (5.09–5.26)	<0.001	1.49(1.46–1.53)
501–1,000	2.97 (2.88–3.06)	<0.001	0.86(0.83–0.89)
1001–1,500	1.76 (1.71–1.88)	<0.001	0.36(0.34–0.38)
1,501–2,000	0.32 (0.27–0.38)	<0.001	0.09(0.08–0.11)
2,001–3,000	0.85 (0.75–0.96)	<0.001	0.24(0.22–0.28)
>3,000	0.71 (0.53–0.92)	<0.001	0.20(0.15–0.27)
NDVI		<0.001‡	
0–0.1§	1.14 (1.06–1.23)		1.00
0.1–0.2	4.21 (4.13–4.29)	<0.001	3.69 (3.42–3.99)
0.2–0.3	4.55 (4.51–4.61)	<0.001	3.99 (3.70–4.30)
>0.3	1.43 (1.36–1.50)	<0.001	1.25 (1.15–1.37)
Precipitation, mm/y		<0.001‡	
0–400§	0.18 (0.14–0.22)	–	1.00
401–800	6.42 (6.34–6.51)	<0.001	36.21 (29.23–45.41)
801–1,200	3.65 (3.58–3.70)	<0.001	20.51 (16.56–25.74)
>1,200	2.64 (2.60–2.69)	<0.001	14.91 (12.03–18.70)
Annual cumulative air temperature, °C		<0.001‡	
0–1,600§	10.18 (9.96–10.39)	–	1.00
1,601–3,400	1.44 (1.38–1.51)	<0.001	0.15 (0.14–0.16)
3,401–4,500	8.01 (7.89–8.12)	<0.001	0.84 (0.82–0.86)
4,501–8,000	2.56 (2.52–2.59)	<0.001	0.27 (0.26–0.27)
>8,000	0.19 (0.10–0.34)	<0.001	0.02 (0.01–0.03)
Land surface temperature, °C		<0.001‡	
<28§	10.75 (10.51–10.99)	–	1.00
28–31	2.62 (2.51–2.73)	<0.001	0.24 (0.23–0.26)
32–34	2.86 (2.79–2.93)	<0.001	0.27 (0.26–0.274)
35–37	4.68 (4.63–4.74)	<0.001	0.43 (0.42–0.45)
>37§	0.98 (0.93–1.03)	<0.001	0.09 (0.09–0.10)

*HFRS, hemorrhagic fever with renal syndrome; CI, confidence interval; NDVI, normalized difference vegetation index. †Incidence = number of HFRS cases/100,000 population. ‡p value of each landscape element; others are p value of subdivision analyses. §Reference group.

The highest HFRS incidence of 6.4/100,000 occurred in the semihumid areas, where precipitation levels are 400–800 mm. The HFRS incidence was ≈50% in areas with precipitation >800 mm. No cases were reported from the arid areas, where the precipitation was <200 mm. The difference in HFRS incidence was statistically significant among different precipitation level (df = 4, p<0.001).

The frigid-temperate zone, with annual cumulative temperature of <1600°C, had the highest HFRS incidence at 10.2/100,000. This was followed by the warm zone (3,400–4,500°C) and semitropical (4,500°C–8,000°C) zones with HFRS incidences of 8.0 and 2.6 per 100,000, respectively. Among different cumulative temperature zone, the HFRS incidences were significantly different (df = 4, p<0.001). There was also a significant difference in HFRS incidence regarding LST (df = 4, p<0.001). The highest incidence of 10.8/100,000 was found in areas with LST <28°C. The incidence dropped when the LST value increased to 28°–34°C and increased again to 4.7/100,000 when LST levels reached 34°–37°C (Table 1).

As to the soil types, the univariate logistic regression analysis showed that anthrosols, alfisol, and semihydromorphic soils, which are good for cultivation, had higher risk for HFRS prevalence. All other soils seemed to be less likely to harbor the disease agent (Table 2).

**Table 2:** Result of univariate logistic analysis in different soil types in relation to HFRS occurrence in China, 1994–1998*

Soil type	p value	OR (95% CI)
Anthrosol	<0.01	1.36 (1.12–1.64)
Ferralisol	<0.01	0.74 (0.61–0.89)
Alfisol	<0.01	1.88 (1.56–2.25)
Semiluvisol	<0.05	0.80 (0.65–0.99)
Caliche	<0.01	0.16 (0.11–0.23)
Arid	<0.01	0.06 (0.03–0.12)
Desert	<0.01	0.42 (0.33–0.54)
Skeletol primitive	<0.01	0.41 (0.33–0.50)
Semihydromorphic	<0.01	2.41 (2.00–2.90)
Hydromorphic	<0.05	0.60 (0.37–0.95)
Saline	<0.01	0.55 (0.39–0.77)
Alpine	<0.01	0.02 (0.01–0.04)

*HFRS, hemorrhagic fever with renal syndrome; OR, odds ratio; CI, confidence interval.

The univariate logistic regression analysis also showed that land for agriculture use, including paddy land, irrigated farmland, nonirrigated farmland, and orchard land, were the landscape elements with high risk for HFRS. Other types of land use, except for timber forest land and wetland, were protective against the disease (Table 3).

**Table 3:** Result of univariate logistic analysis in different land use types in relation to HFRS occurrence in China, 1994–1998*

Land-use type	p value	OR (95% CI)
Rice land	<0.01	1.75 (1.46–2.09)
Irrigated farmland	<0.01	1.49 (1.25–1.77)
Nonirrigated farmland	<0.01	2.39 (1.93–2.97)
Timber forest land	0.63	1.05 (0.86–1.27)
Orchard land	<0.01	2.68 (1.67–4.41)
Sparse woods	<0.01	0.63 (0.51–0.75)
Bush	<0.01	0.52 (0.44–0.62)
Prairie and grassland	<0.01	0.14 (0.11–0.18)
Hilly/mountainous grassland	0.7	0.96 (0.81–1.15)
Desert	<0.01	0.20 (0.14–0.28)
Wetland	<0.05	1.70 (1.02–2.86)
Saline-alkali land	<0.01	0.25 (0.14–0.43)
Bare land	<0.01	0.05 (0.03–0.09)

*OR, odds ratio; CI, confidence interval.

Multivariate logistic regression analysis indicated that elevation, NDVI, precipitation, and annual cumulative temperature were significantly associated with HFRS incidence. Semihydromorphic soils (OR = 1.53), timber forest land (OR = 2.04), and orchard land (OR = 1.97) were risk factors for HFRS incidence (Table 4).

**Table 4:** Result of multivariate logistic regression analysis in relation to HFRS occurrence in China, 1994–1998*

	p value	OR (95% CI)
Elevation, m		
<100	–	1.00
100–200	0.75	0.93 (0.61–1.43)
201–500	0.47	0.87 (0.59–1.28)
501–1,000	<0.01	0.58 (0.39–0.86)
1,001–1,500	<0.01	0.27 (0.17–0.43)
1,501–2,000	<0.01	0.22 (0.12–0.39)
2,001–3,000	<0.01	0.31 (0.16–0.60)
>3,000	<0.01	0.05 (0.01–0.25)
NDVI	<0.01	
<0.1	–	1.00
0.1–0.2	0.73	1.12 (0.60–2.11)
0.2–0.3	0.25	1.44 (0.77–2.69)
>0.3	0.22	0.64 (0.32–1.29)
Precipitation, mm/y	<0.01	
<400	–	1.00
400–800	<0.01	9.94 (3.92–25.23)
801–1,200	<0.01	8.16 (2.97–22.44)
>1,200	<0.01	4.95 (1.70–14.39)
Annual cumulative air temperature, °C	<0.01	
<1,600	–	1.00
1,600–3,400	<0.01	0.47 (0.28–0.79)
3,401–4,500	0.41	1.25 (0.73–2.15)
4,501–8,000	0.17	1.58 (0.82–3.07)
>8,000	0.14	2.76 (0.71–10.72)
Soil or land-use type		
Ferralisol	<0.01	0.65 (0.46–0.90)
Desert	<0.01	0.59 (0.41–0.84)
Skeletol primitive	<0.01	0.66 (0.50–0.88)
Semihydromorphic	<0.01	1.53 (1.14–2.06)
Alpine	<0.01	0.23 (0.07–0.73)
Timber forest	<0.01	2.04 (1.48–2.81)
Orchard	<0.01	1.97 (1.18–3.29)
Sparse woods	<0.01	0.60 (0.46–0.78)
Bare land	0.02	0.45 (0.23–0.87)

*HFRS, hemorrhagic fever with renal syndrome; NVDI, normalized difference vegetation index; OR, odds ratio; CI, confidence interval.

## Discussion

In the early 1990s, the spatial distribution of HFRS and its variation regarding to geographic and meteorologic features were well described in China, based on a national investigation (*18*). However, because of the limitation of technique used in the analyses of that study, the HFRS distribution and related environmental factors could be neither displayed at the county level nor visualized on a digital map. Recently, we used GIS-based spatial analysis to elucidate temporal and spatial distribution of HFRS and to highlight geographic areas with a substantially high incidence of the disease (*12*). The results indicated that the application of GIS, together with spatial statistical techniques, provides ways to quantify explicit HFRS and to further identify environmental factors responsible for the increasing disease risk. In the current study, we combined a landscape epidemiologic approach with GIS and remote sensing techniques to increase our understanding of HFRS and its relationship with landscape elements in China.

HTNV and SEOV, the major causative agents of HFRS in mainland China, are associated with 2 distinct rodent hosts, i.e., *A*. *agrarius* and *R. norvegicus*, respectively. The former thrives in rural areas, while the latter is an anthropophilic urban species. HTNV- and SEOV-related HFRS cases should be differentiated to explore the association between HFRS incidence and landscape elements because each rodent species has its own breeding sites with special landscape attributes. Unfortunately, in China, the reported HFRS cases are not distinguished by causative HV. Since the rodent host (*A. agrarius)* of HTNV usually lives in rural areas, large cities and counties with population density >1,000/km^2^ were excluded from the analyses to remove most, if not all, HFRS cases caused by SEOV and to restrict the study to mainly HTNV-type infections.

The reason for the increased risk for HFRS in regions with lower elevation is not clear; population density and human activities are likely explanations. Population density remarkably increases as elevation decreases and most likely facilitates transmission of HV from rodent hosts to human, subsequently leading to increases in HFRS incidence.

HFRS incidence was highest in the frigid-temperate zone, mostly in northeastern China, followed by incidence in the warm-temperate zone. We assume that the HTNV rodent hosts prefer the temperate area. Very few cases occurred in areas that were either extremely cold or extremely hot. The findings of a previous study on rodent surveillance supported our hypothesis, which suggested that the density as well as HTNV infection rate of *A. agarius* in temperate zones was much higher than those in other areas (*8*)*.*

Economic activities are probable reasons for higher HFRS in the areas of particular soil type and land use. In China, semihydromorphic soil is the major cultivated soil type, usually used for growing wheat, corn, and other crops, which can provide adequate food for rodent hosts and subsequently lead to increase rodent density.

Timber forest and orchard land were also appropriate environments for rodent hosts. Forest workers and farmers had more chances to come into contact with contaminated urine and feces of rodents infected with HTNV. An investigation conducted on various land types showed that the highest trap-success rate of *Apodemus* rodents in the country was 28.9% in Heihe County. The county has 38% timber forestland, 16% nonirrigated farmland, and 3% wetland (H. Chen, pers. comm.).

This study characterized the landscape attributes that seem to be favorable for HFRS incidence. Although analyses are still preliminary, the findings can be helpful for generating hypothesis for further investigation. For better analyses, the human and rodent HFRS surveillance in China, including discrimination of HFRS cases due to different HVs, should be enhanced.
